# Stromal versus tumoral inflammation differentially contribute to metastasis and poor survival in laryngeal squamous cell carcinoma

**DOI:** 10.18632/oncotarget.23865

**Published:** 2018-01-03

**Authors:** Benedikt Höing, Oliver Kanaan, Petra Altenhoff, Robert Petri, Kruthika Thangavelu, Anke Schlüter, Stephan Lang, Agnes Bankfalvi, Sven Brandau

**Affiliations:** ^1^ Department of Otorhinolaryngology, Head and Neck Surgery, University Hospital Essen, Essen, Germany; ^2^ Institute of Pathology, University Hospital Essen, Essen, Germany

**Keywords:** cancer-related inflammation, leukocytes, tumor stroma, head and neck cancer, nodal metastasis

## Abstract

In solid tumors the biology and clinical course are strongly influenced by the interaction of tumor cells and infiltrating stromal host cells. The aim of this study was to assess the relative importance of stromal vs. tumoral inflammation for metastasis and survival in patients with laryngeal squamous cell carcinoma (LSCC).

In 110 patients with tissues from histologically proven LSCC the expression of CD45, CD11b, CD3, MMP-9 and COX-2 was semiquantitatively analyzed in stromal regions and tumor nests.

CD45, CD11b, CD3 and MMP-9 positive cells were more abundant in stroma whereas COX-2 was predominantly expressed in epithelial tumor nests. High expression of stromal CD45 and CD11b on immune cells in tumor regions correlated with COX-2 expression on tumor cells. High levels of CD45 in stroma as well as CD11b and COX-2 in tumor nests were associated with increased metastasis. In contrast, high frequencies of CD3 cells in the tumor core area were associated with reduced metastasis. Overall survival was reduced in patients with high stromal CD45, high tumoral CD11b and high tumoral COX-2 expression.

This is the first study which separately analyzes peritumoral stroma and tumor core area in laryngeal squamous cell carcinoma in terms of CD45, CD11b, CD3, MMP-9 and COX-2 expression. Our results indicate that stroma and tumor islands need to be considered as two separate compartments in the inflammatory tumor microenvironment. Inflammatory stromal leukocytes, abundant myeloid cells in tumor regions and high expression of COX-2 on tumor cells are linked to metastatic disease and poor overall survival.

## INTRODUCTION

Head and neck squamous cell carcinoma (HNSCC) is the sixth most common type of cancer worldwide with well-defined risk factors such as alcohol or tobacco abuse. Nevertheless, no substantial improvement of the patients´ 5-years survival rate over the past decades was achieved [1, 2]. Thus there is demand for intensive research to identify specific histological and molecular patterns of HNSCC to enable development of targeted therapy strategies, which better take into account the biology of the disease.

In many solid tumors, including HNSCC, a proinflammatory tumor microenvironment leads to progression and invasion [3]. Mechanistically, necrotic cell death and upregulated protumoral cellular pathways promote the production of proinflammatory mediators and growth factors; this includes matrix metalloproteases (MMP), VEGF or cyclooxygenase, which are produced by different types of immune and tumor cells [3, 4]. This enables the tumor to invade and proliferate by degrading extracellular matrix or by forming new blood vessels [5]. On the other hand, immune cells may also limit tumor progression and can have substantial anti-tumor activity. Recent metaanalyses have tried to decipher the overall impact of various types of immune cells on cancer prognosis [6]. While these large-scale studies are useful in determining the overall prognostic impact of certain immune cells on disease outcome, they do not always take into account the spatial distribution of immune cells in the tumor microenvironment. Therefore, in this study, we employed five simple and well established biomarkers and determined the correlation of stromal versus tumoral inflammation with metastatic disease in laryngeal squamous cell carcinoma (LSCC).

CD45, also known as PTPRC (protein tyrosine phosphatase receptor type C), is a membranous signaling molecule with extra- and intracellular domains that has various functions in cell cycle including growth regulation, differentiation and cytokine receptor signaling [7]. Initially it was called leukocyte common antigen as it is located on all types of leukocytes with distinct staining patterns [8]. In our study CD45 serves as a panleukocytic cellular marker assessing the immune cell infiltration of stroma and tumor. In order to distinguish between a myeloid and lymphoid infiltrate two further leukocyte lineage markers were applied. CD11b is an adhesion molecule with various functions in the immune system such as phagocytosis and plays a role in the complement system. It is also called Integrin α M and is mainly expressed on myeloid cells such as monocytes, macrophages and granulocytes including neutrophils [9]. CD3 is part of the T-cell receptor complex and thus contributes to T-cell differentiation. It serves as a T-cell marker in our immunohistochemistry [10].

MMP-9 as part of the matrix metalloprotease family is a zinc-dependent protease released by various cell types including endothelial and immune cells as well as fibroblasts to degrade extracellular matrix components. In healthy tissue its function comprises angiogenesis and tissue repair. It is hypothesized that the process of proteolysis and remodeling of matrix components such as collagen type IV (a major component of the basal membrane) facilitates tumor invasion and metastasis [11]. Recently published data underscore the importance of MMP-9 in laryngeal carcinogenesis and progression [12]. In our study MMP-9 serves as a cellular inflammatory marker.

COX-2 is an isoform of the cyclooxygenase enzyme which catalyzes the synthesis of prostaglandins and thromboxane from membranous arachidonic acid. In contrast to COX-1, COX-2 is mainly expressed in an inflammatory environment. COX-2 is upregulated in head and neck cancer [13] and promotes angiogenesis and metastasis [14]. In our study COX-2 serves as an enzyme indicating an inflammatory environment in stroma and tumor.

Histopathologically LSCC consists of tumor and peritumoral stroma each of them having distinct biological characteristics. Previous studies on HNSCC-related inflammation did not separately investigate the inflammatory activity in those two compartments [12, 14, 15]. We hypothesized that inflammatory activity in stromal regions versus tumor nests may have different effects on disease progression and outcome.

In order to test this hypothesis, we assessed the relative importance of stromal versus tumoral inflammation for metastasis and overall survival in laryngeal squamous cell carcinoma applying five inflammatory markers: CD45, CD11b, CD3, MMP-9 and COX-2.

## RESULTS

### CD45, CD11b, CD3, MMP-9 and COX-2 are differentially expressed between stroma and tumor

**Figure 1:**
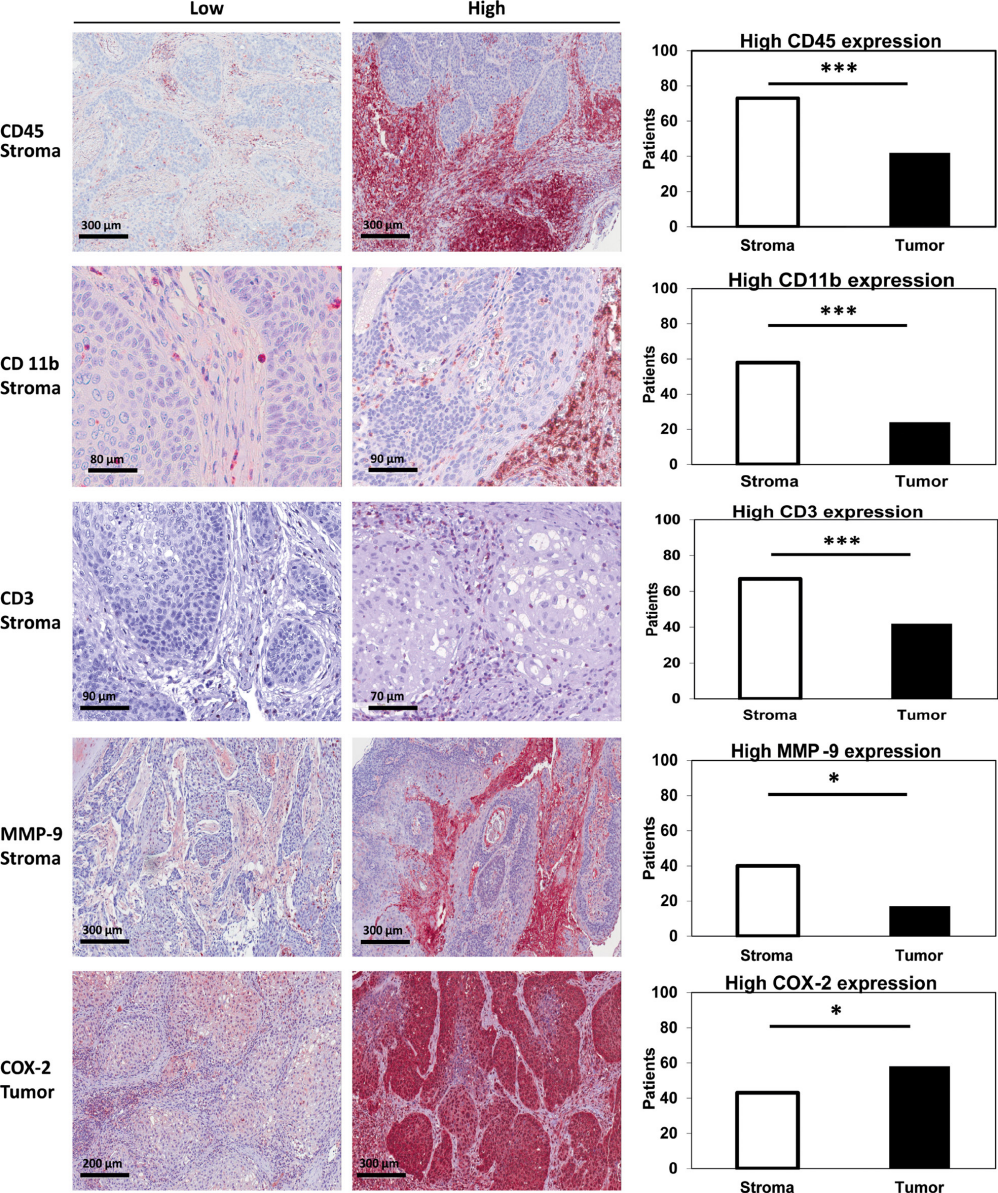
Stromal vs. tumoral expression of CD45/CD11b/CD3/MMP-9 and COX-2 LSCC TMAs were stained for CD45 (1st row), CD11b (2nd row), CD3 (3rd row), MMP-9 (4th row) and COX-2 (5th row). Staining examples (20-40-fold magnification) of low and high scoring are shown for CD45, CD11b, CD3 and MMP9 in stroma as well as for COX-2 in tumor nests. The number of patients with high scores in stroma and tumor are compared by chi-square-test. For all tests, the level of significance was set at *p* < 0.05. ^*^indicates *p* < 0.05, ^***^indicates *p* ≤ 0.001.

Expression of CD45, CD11b, CD3, MMP-9 and COX-2 in stroma and tumor was determined analyzing tissue microarrays of 110 patients with LSCC. In order to detect potential differences in expression patterns, expression was quantitated and compared between stroma and tumor. Whereas 73 patients (66 %) had a high CD45 score in stroma, only 42 patients (38,2 %) showed a high CD45 score in tumor regions (*p* < 0.001, chi-square). Consistently, 58 patients (56,3 %) had a high CD11b score in the stroma whereas only 24 patients (23,3 %) showed a high score in the tumor (*p* = 0.001, chi-square). High CD3 expression was observed in 67 patients (64,4 %) in stroma and in 42 patients (40,4 %) in the tumor (*p* < 0.001, chi-square). Regarding MMP-9, 40 patients (37 %) had a high score in stroma compared to 17 patients (15,7 %) with a high score in tumor regions (*p* = 0.036, chi-square). In contrast, high expression of COX-2 was more frequently observed in tumor nests (*n* = 58, 53 %) as compared to stromal regions (*n* = 43, 39 %; *p* = 0.021, chi-square, Figure 1).

### Correlation analysis

**Figure 2:**
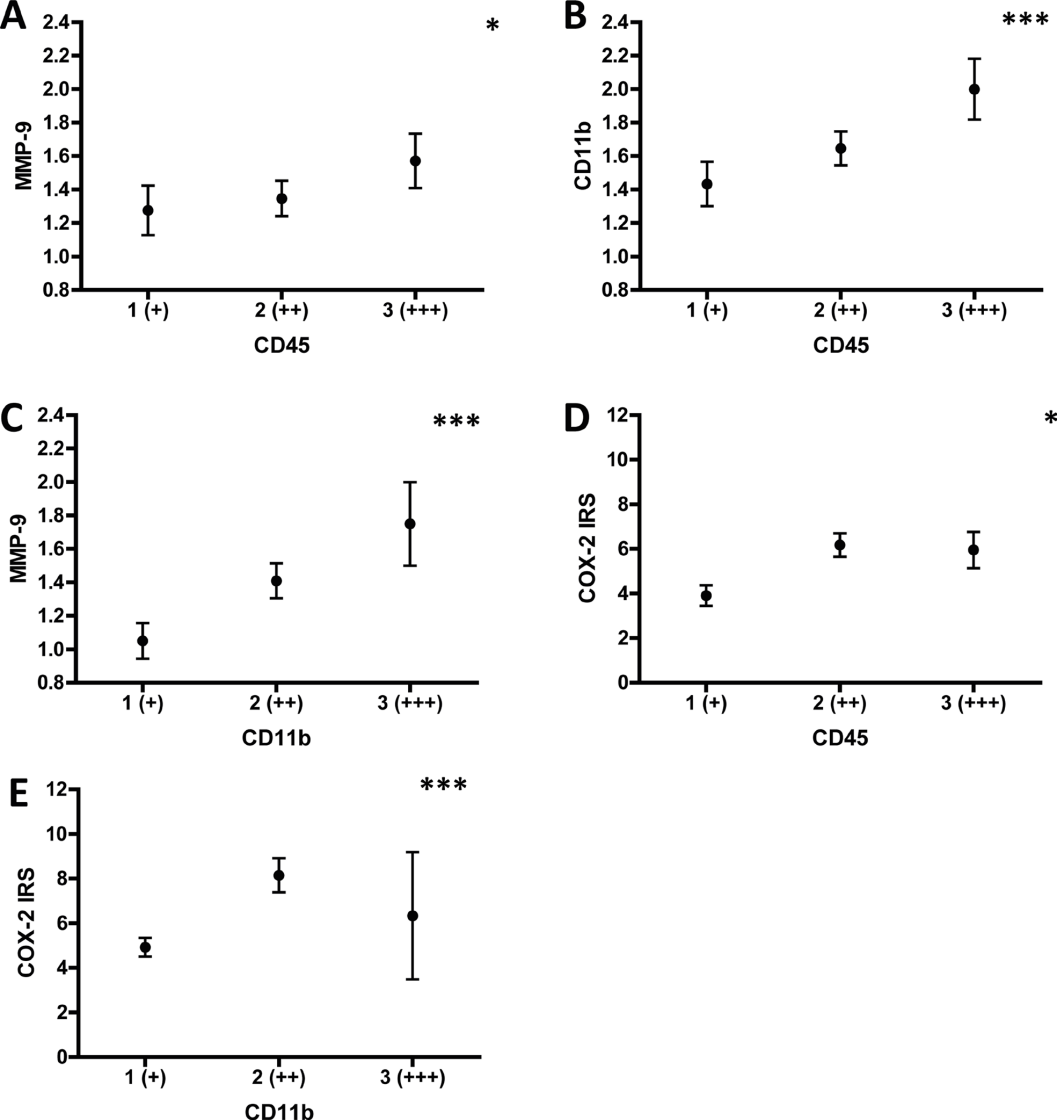
Correlation analysis of stromal CD45/CD11b/CD3/MMP-9 and tumoral CD11b/COX-2 expression Expression of stromal CD45, CD11b, CD3, MMP9 and tumoral CD11b, COX-2 was determined as illustrated in Figure 5. Correlations between the markers in the respective patient groups were calculated using Spearman-Rho. (**A**) Means of stromal MMP-9 expression were calculated by using 1 (+), 2 (++) or 3 (+++) and plotted for each stromal CD45 expression category. (**B**) Means of stromal CD11b expression were calculated by using 1 (+), 2 (++) or 3 (+++) and plotted for each stromal CD45 expression category. (**C**) Means of stromal MMP-9 expression were calculated by using 1 (+), 2 (++) or 3 (+++) and plotted for each stromal CD11b expression category. (**D**) Means of tumoral COX-2 expression are indicated by the IRS (range 0-12) and plotted for each stromal CD45 expression category. (**E**) Means of tumoral COX-2 expression are indicated by the IRS (range 0-12) and plotted for each tumoral CD11b expression category. ^*^indicates *p* < 0.05, ^***^indicates *p* ≤ 0.001.

Next, we determined potential correlations between CD45, CD11b, CD3, MMP-9 and COX-2 expression in stroma and tumor, respectively. We found a significant positive correlation between stromal CD45 and stromal MMP-9 expression (*r* = 0.207, *p* = 0.032, Figure 2A). Of note, the number of CD45 positive cells in the stroma also correlated with the frequency of stromal CD11b (*r* = 0.317, *p* = 0.001, Figure 2B), but not with CD3 positive cells (*r* = 0.094, *p* = 0.344). Stromal CD11b (*r* = 0.314, *p* = 0.001, Figure 2C), but not CD3 (*r* = 0.161, *p* = 0.104) expression positively correlated with stromal MMP-9 expression. Interestingly, stromal CD45 also positively correlated with tumoral COX-2 expression (*r* = 0.215, *p* = 0.025, Figure 2D). Correlating tumoral CD45 and tumoral MMP-9 expression, the *p*-value was indicative of a borderline significance (*r* = 0.19, *p* = 0.051). Importantly, the frequency of tumoral CD11b positive cells highly correlated with tumoral COX-2 expression (*r* = 0.347, *p* < 0.001, Figure 2E). No significant correlations were found for tumoral CD45 and tumoral COX-2 expression (*r* = 0.032, *p* = 0.743), and tumoral MMP-9 and tumoral COX-2 expression (*r* = 0.119, *p* = 0.219). Stromal expression of CD45 did not correlate with stromal COX-2 expression (*r* = 0.081, *p* = 0.4), neither did stromal COX-2 with stromal MMP-9 (*r* = 0.035, *p* = 0.722). Of note, COX-2 tumoral staining intensity was highly correlated with the number of COX-2 positive tumor cells (*r* = 0.567, *p* < 0.001). These data highlight that tumor-infiltrating leukocytes and the pro-metastatic enzyme MMP-9 are preferentially expressed in stromal regions of the tumor tissue, whereas COX-2 is more prominently expressed in carcinoma cells in tumor nests. Moreover, potential connections between stromal total leukocytes (CD45) and tumor nest infiltrating myeloid cells (CD11b) with expression of COX-2 in tumor cells are established.

### Stromal CD45 and COX-2 expression by tumor cells are linked to lymph node metastasis

**Figure 3:**
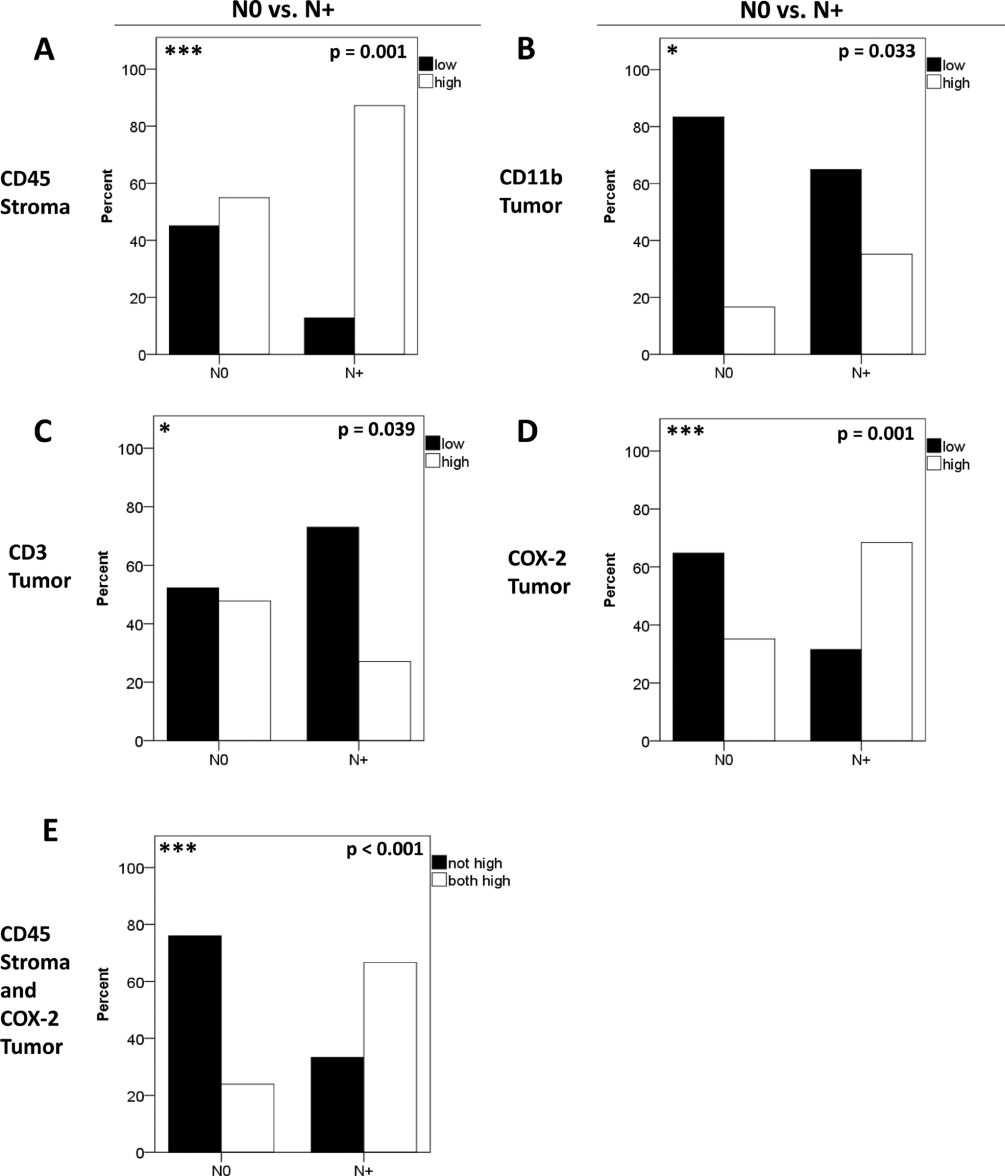
Correlation of biomarkers with nodal metastasis Patients were separated according to their N-status in N+ and N0. The distribution of stromal CD45 (**A**), tumoral CD11b (**B**), tumoral CD3 (**C**) and tumoral COX-2 (**D**) expression is shown for N+ versus N0. Differences in expression patterns between groups were calculated using chi-square-test. Combining high stromal CD45 and tumoral COX-2 expression (**E**) revealed the most significant association with patients´ N-status (*p* < 0.001). Patients with simultaneous high stromal CD45 and high tumoral COX-2 expression were labeled “both high”.

In order to test whether inflammatory infiltrates and markers are linked to metastasis we tested for an association of marker expression with N-stage. To this end patients with (N+) or without (N0) nodal metastases were compared with respect to the expression of CD45/CD11b/CD3/MMP-9/COX-2. The majority of patients with positive nodal status displayed high stromal CD45 expression (*p* = 0.001, chi-square, Figure 3A). Interestingly, metastasized patients (N+) displayed higher tumoral CD11b frequencies than N0 patients (0.033, chi-square, Figure 3B) whereas high tumoral CD3 expression was more frequently observed in N0 patients (*p* = 0.039, Figure 3C). In contrast, stromal MMP-9 was not correlated with N-status (*p* = 0.901, chi-square). Interestingly, the N+ group contained significantly more patients with a high tumoral COX-2 expression (*p* = 0.001, chi-square, Figure 3D). Combining high stromal CD45 and tumoral COX-2 expression revealed the most significant difference between patients with N0 and N+ status (*p* < 0.001, chi-square, Figure 3E). No significant differences between groups (N0 vs. N+) were obtained for tumoral CD45 (*p* = 0.12), stromal CD11b (*p* = 0.73), stromal CD3 (*p* = 0.720), tumoral MMP-9 (*p* = 0.228) and stromal COX-2 (*p* = 0.221, data not shown).

### High stromal infiltration by leukocytes correlates with poor overall survival

**Figure 4:**
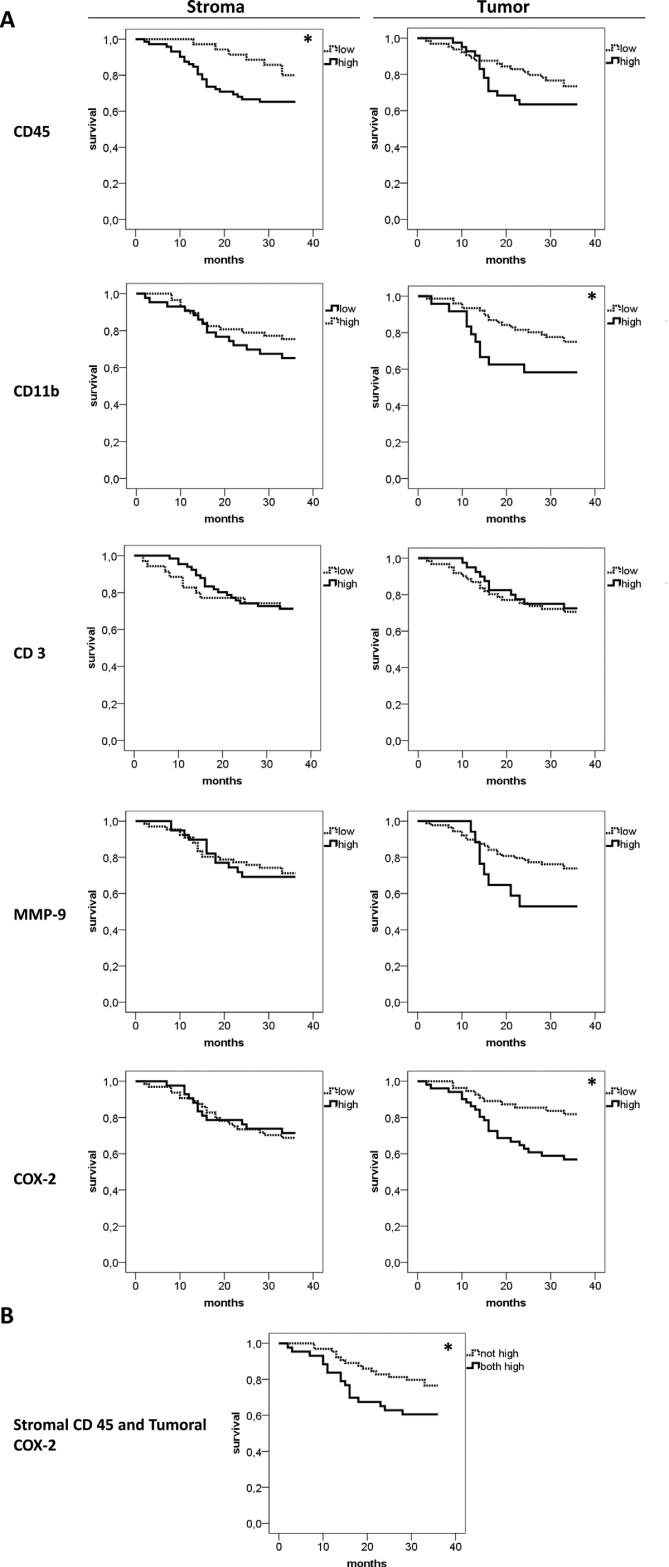
Comparative 3-years survival analysis of stromal and tumoral inflammation Overall 3-years survival analysis for CD45, CD11b, CD3, MMP-9 and COX-2 was performed separately for stroma and tumor (**A**) High CD45 expression in the stroma, but not in the tumor (first row), was associated with lower survival (*p* = 0.047). In contrast, high CD11b infiltration in the tumor nests, not in the stroma, showed an association with lower survival (*p* = 0.043). CD3 and MMP-9 expression had no significant influence on patients´ survival. Interestingly, high COX-2 expression in the tumor, not in the stroma, was associated with a significantly lower survival rate (*p* = 0.009). (**B**) The combination of high stromal CD45 and high tumoral COX-2 was associated with earlier death (*p* = 0.037). For this analysis patients with simultaneous high stromal CD45 and high tumoral COX-2 expression were included in the “both high” group.

Lastly the impact of stromal and tumoral inflammation on patients´ survival was analyzed (Figure 4). High stromal CD45 (*p* = 0.047) expression was associated with reduced 3-year overall survival rate. By tendency the frequency of CD45 positive cells in epithelial tumor nests was also associated with survival, but data clearly failed to reach statistical significance. (*p* = 0.244). On the other side, high tumoral, but not stromal CD11b expression was associated with reduced 3-year overall survival rate (*p* = 0.043) For CD3 and MMP-9 neither stromal (*p* = 0.738 for CD3; *p* = 0.915 for MMP-9) nor tumoral (*p* = 0.675 for CD3; *p* = 0.097 for MMP-9) expression correlated with overall survival rates. Of note, high tumoral (*p* = 0.009) but not high stromal COX-2 (*p* = 0.815) expression is associated with impaired overall survival. Combining high stromal CD45 and tumoral COX-2 also showed a significantly lower 3-years survival rate (*p* = 0.037, Figure 4B). As the correlation analysis revealed a borderline result for tumoral CD45 and tumoral MMP-9 (*p* = 0.051), survival analysis was calculated combining high tumoral CD45 and MMP-9. However, for this combination, no significant effect on survival was found (*p* = 0.107).

In a final step we included possible confounding factors such as the treatment modality, extracapsular lymph node spread, T- and N-stage in a multivariate regression analysis model. In this analysis the negative prognostic impact of stromal CD45, CD11b+ cells in tumor nests and combination of CD45/tumoral COX-2 was independent of all tested confounders (*p*-values ranging from 0.091 to 0.871) and thus remained significant in this multivariate analysis. Correlation of tumoral COX-2 with survival was independent of T-stage (*p* = 0.612) but confounded by N-stage (*p* = 0.03).

All in all, our data suggest a differential and specific contribution of stromal versus tumoral inflammatory mediators to tumor progression and reduced survival in LSCC.

## DISCUSSION

This is the first study separately analyzing, for stroma and tumor cell areas, the expression of important cellular and soluble inflammatory mediators in a large cohort of patients with LSCC. The aim was to test whether these markers of cancer-related inflammation are associated with survival and metastatic state and whether a potential association differs between inflammation localized in stromal regions versus tumor islands.

Our results show that CD45-positive leukocytes and their subsets, CD11b-positive and lymphoid (CD3) cells, are mainly localized in the stroma. Strong stromal leukocytic and tumoral CD11b infiltration are associated with higher N-stage and reduced 3-years overall survival rate. High frequencies of T cells in tumor nests are associated with the absence of lymph node metastses. Similarly, also MMP-9 was mainly expressed in the stroma and correlated with stromal CD45. However, unlike CD45, the frequency of MMP9-positive cells in the stroma was not related to metastasis and survival. In contrast to the other markers, COX-2 was mainly expressed in the tumor nests. Also expression of COX-2 in the tumor cells was significantly associated with higher N-status and lower survival. These findings are in agreement with previous results suggesting a crucial role for COX-2 in metastasis of HNSCC [13, 14]. Tumoral CD45, stromal CD11b, CD3 and stromal COX-2 were not associated with lymph node metastasis and overall survival. Thus, our results highlight the importance of the localization of inflammatory mediators and circuits within the complex tumor microenvironment.

Cancer-related inflammation and the expression of related biomarkers are now well recognized as important players in tumor progression [5, 16, 17]. Consequently, these markers became attractive prognostic or even predictive biomarkers [18, 19]. In oral and laryngeal squamous cell carcinoma high expression of COX-2 was related to metastasis [14] and reduced survival [15]. A recent study addresses the tumor microenvironment of HNSCC focusing on immune cell infiltration with a low neutrophil-to-lymphocyte ratio predicting a better response to induction chemotherapy [20] which is in accordance with our data that suggest a positive effect of a high tumoral T-cell infiltrate on control of metastasis and an adverse prognostic effect of high tumoral CD11b expression.

In a more recently developed concept the localization of immune cells has been used as a major descriptor of the tumor microenvironment. In this concept, depending on the T cell frequency and localization, immune desert, T cell inflamed or T cell excluded tumors have been described [21]. It has also been shown that these categories have implications for response to immunotherapy [22], and in particular checkpoint blockade [23]. Interestingly, we show here that tumor cell expression of COX-2 correlates with stromal CD45 (total leukocytes) and expression of the myeloid marker CD11b in tumor cell nests. These data suggest that expression of COX-2 in tumor cells influences the recruitment of immune cells into the tumor tissue and at least partially instructs the “immune phenotype”. However, COX-2 expression did not correlate with CD3 counts, suggesting that other factors than COX-2 modulate the T-cell infiltrate in LSCC.

T-cells represent a major arm of antitumor immunity leading to better prognosis and outcome in various cancer types including head and neck cancer [24, 25]. Indeed we found that a low T-cell infiltrate in tumor nests was associated with higher rates of lymph node metastasis in our study. These data suggest that in LSCC T-cells could be involved in control of metastasis. In accordance, a high lymphoid CD8+ T-cell infiltrate in patients with carcinomas of oro- and hypopharynx [26] and a high tumoral T-cell expression in breast cancer cells [27] were shown to have positive prognostic effects. Initially, in our study, we hypothesized that the correlation of high stromal CD45 infiltration and patients´ survival rates might be due to stromal T-cell infiltration. However, our analyses did not support this notion and stromal leukocyte subsets explaining the correlation of high stromal CD45 with survival still need to be identified in future studies.

As many ongoing and recent studies still do not consider compartmentalization of tumor tissues, a major implication of our study is to consider the exact localization of immune and cell biological biomarkers within the tumor microenvironment as an intensive cross-talk between infiltrating immune cells and tumor cells exists [28]. Consistently, our study suggests that stroma and tumor are distinct, however interacting, compartments in the tumor microenvironment of LSCC exemplified by correlations of stromal expression of CD45 with the frequency of tumoral CD11b as well as tumoral COX-2 expression. Our data are in accordance with a hypothesis in which either tumor mediators such as COX-2 instruct the influx of immune cells or alternatively incoming immune cells induce the further activation of tumor cells [28, 29]. Previous studies already highlight the importance of interactions between tumor cells and infiltrating immune cells in various cancer types focusing on COX-2 as an inflammatory mediator [30, 31]. For example, a recent study demonstrated that direct cell-cell interactions between immune cells (neutrophils) and tumor cells lead to enhanced levels of COX-2 with increased tumor growth [32] supporting the theory of interacting stromal and tumoral compartments in the tumor microenvironment.

This study used tissue microarrays of tumor samples for the immunohistochemical analysis. While this is a very common technology to date, it is also important to note that it has certain inherent limitations and caveats with respect to tumor heterogeneity and composition of the TMA analyzed. Intratumoral heterogeneity concerning immune cell distribution between tumor core and tumor margin is well known [33]. Besides that, two other types of intratumoral heterogeneities exist. Firstly, regarding the whole tumor, there might be an uneven distribution of immune cells resulting in a patchy staining pattern with accumulation of cells in hot spots. Secondly, a given heterogeneity induced by different local stroma-tumor ratios is likely to be observed. In an attempt to measure and verify the heterogeneity in this study, intrapatient variability in three fields of view for CD3 and CD11b was compared among each other and to interpatient variability separately for stroma and tumor. Considering the results of the performed analysis of variance showing a consistently low intrapatient variability across groups which itself is lower than interpatient variability, we believe that, despite certain caveats, a valid scoring system was applied in this study.

Taken together our results provide first evidence for the existence of distinct inflammatory mediators associated with tumor progression and disease outcome in LSCC. These mediators can have a location-specific distribution between tumor nests and peritumoral mesenchymal stroma. We suggest that future studies should more comprehensively take into account the cell-type- and localization-specific expression of promising biomarkers. Together with the increasing knowledge on the biological interactions in the complex tumor microenvironment this will guide the development of meaningful and clinical relevant biomarkers in the future.

## MATERIALS AND METHODS

### Clinicopathological data and study subjects

Tissue microarrays (TMAs) from 110 patients with histologically proven laryngeal squamous cell carcinoma were included in the study. All patients were treated at the Department of Otorhinolaryngology and tumor sections were assessed at the Institute of Pathology, University Hospital Essen, between 1995 and 2005. Clinical follow-up data was obtained over 3 years. Patient characteristics are shown in Table 1. Experiments were approved by the local ethics committee and conducted according to the Declaration of Helsinki.

**Table 1:** Clinical data of 110 patients with laryngeal squamous cell carcinoma (LSCC)

		Count	% of total	Mean
**Gender**	male	96	87,0%	
	female	14	13,0%	
**Age**				60 (SD 9,92)
**T-stage**	T1	23	20,9%	
	T2	32	29,1%	
	T3	25	22,7%	
	T4	30	27,3%	
**N-stage**	N0	71	64,5%	
	N1	11	10,0%	
	N2a	0		
	N2b	13	11,8%	
	N2c	14	12,7%	
	N3	1	1,0%	
**N0 vs. N+**	N0	71	64,5%	
	N+	39	35,5%	
**M-stage**	M0	108	98,2%	
	M1	2	1,8%	
**Histopathological Grading**	G1	6	5,5%	
	G2	80	72,7%	
	G3	21	19,1%	
	unknown	3	2,7%	
**Extracapsular lymph node spread**	ECE	3	7,7%	
	No ECE	29	74,4%	
	unknown	7	17,9%	
**Therapy**	surgery	49	44,5%	
	surgery + adjuvant RTX	42	38,1%	
	surgery + adjuvant RCTX	16	14,4%	
	RCTX	1	1,0%	
	RTX	1	1,0%	
	RCTX + salvage surgery	1	1,0%	

ECE = extracapsular extension, RTX = radiotherapy, RCTX = radiochemotherapy.

### Immunohistochemical staining

**Figure 5:**
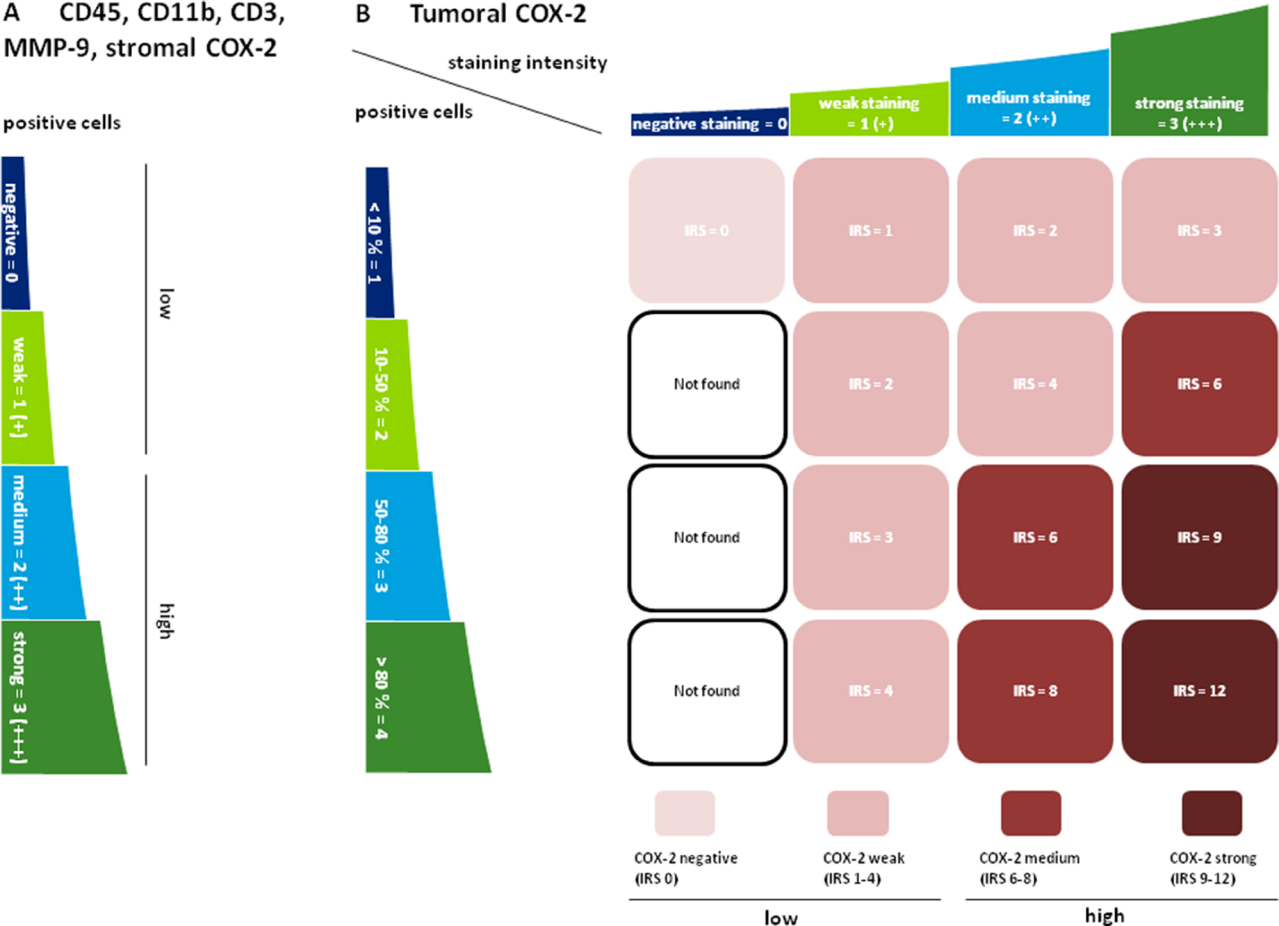
Semiquantitative scoring of CD45 / CD11b / CD3 / MMP-9 and COX-2 (**A**) The infiltration of tumoral and stromal regions with CD45, CD11b, CD3 and MMP-9 positive cells as well as the infiltration of stroma with COX-2 positive cells was labeled negative (0), weak (+), medium (++) or strong (+++). Negative and weak scores were categorized as “low” whereas medium and strong staining were subsumed as “high”. For CD3, only patients with a negative scoring were included in the “low” group. This was due to the fact that most patients were in the negative or (+) group and only a very small number of patients (*n* = 8) presented with a medium (++) or strong (+++) intratumoral score (**B**) COX-2 was predominantly expressed in the tumor cells and varied in terms of staining intensity. Therefore an immunoreactive score (IRS) for tumoral COX-2 was calculated by multiplication of positive tumor cells with staining intensity. IRS < 6 (0–4) were labeled „low“, IRS ≥ 6 (6-12) indicated “high” expression levels.

Tissue microarrays of LSCC patients were prepared and 3 µm sections were cut. Next, slides were deparaffinized and antigens were retrieved by HIER (heat-induced epitope retrieval) in citrate buffer pH 6.0 (Invitrogen, Karlsruhe, Germany) boiling for 15 minutes. The primary antibodies monoclonal mouse anti-human CD45 (Sigma-Aldrich, Taufkirchen, Germany, dilution 1:500), polyclonal rabbit anti-human CD11b (Sigma-Aldrich, Taufkirchen, Germany, dilution 1:300), monoclonal rabbit anti-human CD3 (RabMAb/abcam, Cambridge, UK) dilution 1:10), polyclonal rabbit anti-human MMP-9 (LifeSpan BioSciences, Seattle, USA, dilution 1:500) and polyclonal goat anti-human COX-2 (Bio-Techne, Wiesbaden-Nordenstadt, Germany, dilution 1:100) were applied at 4°C overnight. Then samples were incubated with horseradish peroxidase (HRP)-coupled secondary antibodies rabbit anti-mouse F(ab’)2, goat anti rabbit F(ab’)2 or donkey anti-goat F(ab’)2 (all Dianova, Hamburg, Germany) for 30 min at room temperature. Colorimetric reactions were done with AEC Single Solution (Invitrogen) for 10 min and nuclei were counterstained with Haematoxylin (Shandon/Thermofisher Scientific, Bonn, Germany) for 1 min. Lastly, the sections were covered with Kaisers Glycerin gelatin (Merck, Darmstadt, Germany). Stained TMAs were digitalized by using Scan Scope CS2 and were analyzed with Aperio Image Scope (both Zeiss, Wetzlar, Germany). 110 tumors were stained with the anti-CD45 antibody, 103 tumors with anti-CD11b, 104 tumors with anti-CD3, 108 tumors with anti-MMP-9 and 109 tumors with anti-COX-2. Quantification of the immunohistochemical stainings was performed using a semiquantitative scoring system, which is illustrated in Figure 5.

### Data and statistical analysis

Blinded scoring was performed independently by two observers under the supervision of A.B. (senior histopathologist). CD45, CD11b, CD3 and MMP-9 as cellular immune cell markers were scored according to the number of positive cells (Figure 5A). For COX-2 an immunoreactive score (IRS) for the tumor islands was used (Figure 5B). Thereby tumoral COX-2 expression was scored regarding the COX-2-specific staining pattern of the tumor cells. Statistical analysis was performed using SPSS Software Version 22 (IBM, Chicago, USA). Descriptive statistics was done before applying non-parametric tests for ordinally scaled data to compare differences between groups. Spearman-rho correlation analysis was performed to unveil associations between expression patterns. Survival data was analyzed at a cut-off point of 3 years after first diagnosis according to the Kaplan-Meier method.

Coefficients of variation for stromal and tumoral CD3 and CD11b were compared applying Levene´s test. It could be shown that intrapatient scoring variability measured by means of intrapatient coefficients of variation did not differ significantly between groups (*p* = 0.648 for tumoral vs. stromal CD3, *p* = 0.687 for tumoral CD3 vs. tumoral CD11b, *p* = 0.923 for tumoral CD3 vs. stromal CD11b, *p* = 0.395 for stromal CD3 vs. tumoral CD11b, *p* = 0.774 for stromal CD3 vs. stromal CD11b and *p* = 0.649 for tumoral vs. stromal CD11b). The mean of intrapatient variability (0.52) was lower than interpatient variability (0.83) with a *p*-value indicating a borderline significant difference (*p* = 0.067). Taken together, these data show low intrapatient variability and validate the scoring system used in this study.
